# Electrothermal bipolar vessel sealing device (LigaSure™) versus conventional diathermy in laparoscopic myomectomy: A propensity-matched analysis

**DOI:** 10.1371/journal.pone.0193611

**Published:** 2018-03-01

**Authors:** Yi-Chieh Li, Angel Chao, Lan-Yang Yang, Hui-Yu Huang, Yi-Ting Huang, Hsin-Hong Kuo, Chin-Jung Wang

**Affiliations:** 1 Department of Obstetrics and Gynecology, Chang Gung Memorial Hospital Linkou Medical Center and Chang Gung University College of Medicine, Taoyuan, Taiwan; 2 Biostatistics Unit, Clinical Trial Center, Chang Gung Memorial Hospital Linkou Medical Center, Taoyuan, Taiwan; 3 Department of Obstetrics and Gynecology, Chang Gung Memorial Hospital at Taipei, Taipei, Taiwan; Chi Mei Medical Center, TAIWAN

## Abstract

The purpose of this study was to compare the safety and efficacy of an electrothermal bipolar vessel sealing device (LigaSure™) and traditional electrical cauterization in laparoscopic myomectomy (LM). A total of 756 patients with symptomatic uterine myomas who underwent LM were reviewed retrospectively. A total of 225 cases of LM using LigaSure™ (LML group) were compared with a control group treated with traditional electrical cauterization (LME group) under propensity-matched analysis. Outcome measures for both groups were compared, such as operative time, blood loss (BL), complications, need for blood transfusion, hospital expenses, and hospital stay. Six subgroups were divided according to main myoma size and energy source. No cases required switching to abdominal myomectomy. The number of myomas removed, BL, need for blood transfusion, and complications were not significantly different, whereas hospital stay was longer in the LME group than in the LML group and total hospital expenses were higher in the LML group (p < 0.001). The overall operation duration was significantly longer in the LML group but was not significantly different for main myoma >10 cm (LML vs LME, 121.58 ± 41.77 vs 121.69 ± 44.95, p = 0.99); this likely reflects the operative efficiency on using LigaSure™ to manage large tumors. Significant linear correlations between myoma weight and operative time and BL were seen in both groups. Conventional diathermy is more effective for small-to-medium myomas. Use of the LigaSure™ was efficient for myomas >10 cm.

## Introduction

Myomectomy is a treatment choice for women with symptomatic uterine leiomyoma who want to keep their uterus. Since its introduction in 1979 [1], laparoscopic myomectomy (LM) has been considered a safe and effective alternative to laparotomy myomectomy. Nonetheless, it is often considered more challenging because of its higher technical requirement for laparoscopic extraction of leiomyoma, primary uterine repair, and specimen removal [2].

The three major steps of LM include uterine myoma excision, uterine defect repair, and surgical specimen extraction [2]. Improvements in the efficacy of each step would contribute to minimizing operative blood loss and operative time. Myoma excision includes an incision in the serosa overlying the myoma, extension of the incision into the pseudocapsule, dissection of the pseudocapsule attachment, and bleeding control for nurturing vessels. All of these steps are time-consuming and prone to bleeding complications and may potentially benefit from the use of more advanced equipment.

Whereas conventional diathermy is reliable for blood vessels >2 mm in diameter, the hemostatic LigaSure™ (Valleylab, Boulder, CO, USA) device seals blood vessels ≤7 mm in diameter. Using an exact amount of bipolar diathermy with physical pressure, LigaSure™ permanently denatures elastin and collagen in the target tissue. Meanwhile, this instrument reduces thermal spread by automatically switching off when the impedance of the targeted tissue reaches a specified limit [3]. Since its introduction in 1998, LigaSure™ has shown equal performance to traditional bipolar instruments for laparoscopic hysterectomy and adnexectomy regarding operative time and blood loss [4, 5]. Nevertheless, its efficacy in LM has not been well established.

A literature search yielded few studies discussing the role of LigaSure™ in LM. In this study, we compare the LM results achieved using LigaSure™ to those on using traditional diathermy in a matched-control group.

## Methods

We retrospectively recruited 225 patients (age range, 24–54 years; mean, 39.0 ± 6.3 years) with different indications who were scheduled to undergo LM with the LigaSure™ bipolar vessel sealing system (LML), performed by one of the authors (C.J.W.), between February 2010 and August 2014 at Chang Gung Memorial Hospital. Five-hundred thirty-one women undergoing LM using conventional electrosurgery (LME), performed by the same operator (C.J.W.), were also retrospectively recruited to avoid technique bias. LigaSure™ was introduced into our hospital in 2010; however, we do not perform LM with the LigaSure™ routinely because its use requires extra charges in accordance with the national healthcare policy in Taiwan. The surgical indications in these women included menometrorrhagia, abdominal-pelvic pain, pelvic pressure (i.e., frequent urination or backache), and infertility. Prior to surgery, all women underwent pre-operative evaluations including pelvic examination, detailed medical history, and ultrasonography. Women with sex experience were screened for cervical cancer. Diagnostic hysteroscopy was performed to exclude pathological lesions in the uterine cavity in patients suffering from menometrorrhagia and anemia. Patients were informed of the surgical risks, including the possibility of converting to laparotomy, intra-operative massive bleeding, need for transfusion, and post-operative adhesion formation. All patients gave their written informed consent, and all received an enema and intravenous prophylactic cephalosporin (1 g).

Data were collected on patient demographics (i.e., age and body mass index), weight of the excised myomas in grams, number of cesarean deliveries, pre-treatment with a gonadotropin-releasing hormone (GnRH) agonist, operative details (i.e., operative time, size and number of myomas removed, blood loss, and need for blood transfusion), and post-operative outcomes (i.e., hemoglobin decrease, hospital stay, and nature of complications). Data on total hospital expenses (the money was not paid by the National Healthcare System) were obtained from the hospital’s electronic medical records. This study was reviewed and approved by the human investigation review board of Chang Gung Memorial Hospital.

### Operative procedures

Operation began under general anesthesia, with the patient in the low lithotomy position and the legs supported in stirrups. A nelaton tube was inserted into the bladder to constantly drain urine. LM was performed per the technique described by Wang et al [6]. Briefly, after adequate pneumoperitoneum was achieved, trocar placement in position, and all the myomas identified, a conventional unipolar was used to transversely incise along the avascular plane overlying the largest myoma until its pseudocapsule was reached. After the cleavage plane was identified, the myoma was pulled hard upward using 5 mm claw forceps or a stainless-steel centimeter probe to apply traction and countertraction pressure. The unipolar electrode and bipolar forceps or LigaSure™ was used to further dissect the pseudocapsule attachments. Additional myomas were removed through the same manner. After enucleation of myomas, bleeding points were identified and controlled with electrical cauterization (bipolar cautery or LigaSure™).

The uterine muscle and serosa were then closed in two layers with a zero monofilament poliglecaprone 25 (Monocryl, Ethicon Inc., Somerville, NJ, USA) continuous non-running-lock suture and intracorporeal knots. Specimens were routinely extracted through the posterior colpotomy. The colpotomy wound was closed with 2–0 polyglycolic acid sutures. For specimens that had to be removed from the abdominal wall (e.g., women without sexual activity), a 15 mm electromechanical morcellator (Ethicon Endosurgery, Cincinnati, OH, USA) was used to ease the extraction. After complete hemostasis was achieved, all cannula sites were closed with 3–0 polyglycolic acid sutures at the level of the fascia. Sterile adhesive tape was used for skin closure.

### Statistical analysis

Student’s *t*-test was used to compare continuous variables, while Pearson’s chi-squared test and Fisher’s exact test were used to compare categorical variables. Multiple logistic regression was used to estimate the likelihood of undergoing a LML for all women according to myoma weight, body mass index (BMI), GnRH agonist pretreatment, age, and number of cesarean deliveries. The logistic model produces a zero to one propensity score based on the predicted probability of undergoing LME versus LML, which relied upon the differences in patient demographics and preoperative distinctive features [7]. Selection bias for receiving LME or LML was then measured using these propensity scores. A matching on the presented propensity score with 1:1 ratio by nearest neighbor approach was used to reduce the selection bias for analysis and comparison between LME and LML.

Six operative outcomes (i.e., number of myomas removed, main myoma size, blood loss, hemoglobin decrease, complications, and need for blood transfusion), and three efficiency outcomes (i.e., hospital expenses, operative time, and post-operative hospital stay) were compared. Pearson’s correlation was used to assess the linear relationship between variables. SPSS for Windows version 18 (SPSS Inc., Chicago, IL, USA) was used for the statistical calculations.

## Results

Patients with LME were younger (mean ± standard deviation,37.6 ± 5.9 vs 39 ± 6.3 years, p = 0.003) and more likely to have smaller myoma weights than those with LML (mean ± standard deviation, 164.7 ± 149.6 vs 233.7 ± 173.5 g, p = 0.005). Patients with LML were more likely to undergo GnRH agonist pretreatment (OR 5.45, 95% CI 3.54–8.41, p <0.001) (**Table 1**). The propensity score model had a classification accuracy (*c* statistic = 0.7) when compared to a strong model of 0.8.

**Table 1:** Comparative patient characteristics for laparoscopic myomectomy with either electrothermal bipolar vessel sealing device (LigaSure™) or electrosurgery logistic regression results for the propensity score model.

	LigaSure™ (n = 225)	Electrosurgery (n = 531)	Odds Ratio (95% CI)	p-value
Fibroid weight (g)	233.71±173.51	164.71±149.57	1.00(1.00–1.00)	0.005
Age (y)	39.00±6.34	37.64±5.93	1.04(1.02–1.08)	0.003
Body Mass Index (kg/m^2^)	22.91±3.60	22.92±3.43	0.99(0.95–1.04)	0.781
Cesarean delivery	0.22±0.57	0.26±0.65	0.93(0.71–1.23)	0.629
Pre-op GnRHa treatment	84(37.33%)	44(8.29%)	5.45(3.54–8.41)	<0.001

Abbreviations: Pre-op, pre-operative; GnRHa, gonadotropin-releasing hormone analogs.

Data are reported as mean (standard deviation) or number (%).

**Table 2**shows the operative outcomes stratified by main myoma size because a high correlation exists between size and weight of the myoma (P < 0.001). The mean amount of blood loss, hemoglobin level decrease, and requirement of blood transfusion did not differ among groups despite myoma size. The mean surgical time was lower in the LME group with a mean myoma size < 10 cm compared to the LML group; however, it did not differ in patients with a large myoma size (≥10 cm) between groups. The mean postoperative hospital stay was lower in the LML group compared to the LME group. On the contrary, hospital expenses were significantly higher in the LML group. Seven women (all from the LME group) suffered from intraoperative bleeding >1000 mL. The extreme bleeding mainly resulted from a large myoma in three women (10, 12, and 12 cm), multiple tumors requiring removal in two women (seven and eight myomas), increased operative difficulty owing to prior laparotomies in one woman, and concomitant suctional curettage for an unwanted pregnancy (11 weeks’ gestation) in one woman. All of them recovered uneventfully after blood transfusion with 4–8 units of packed red blood cells and whole blood, as well as intravenous administration of 1 g cefamezine every 6 h for 2 days.

**Table 2:** Outcomes per main fibroid size category for laparoscopic myomectomy with electrothermal bipolar vessel sealing device (LigaSure™) versus with electrosurgery.

	Small fibroid size, ≤ 6 cm			Intermediate fibroid size, 6–10 cm			Large fibroid size, ≥ 10 cm		
	LigaSure™ (n = 45)	Electrosurgery (n = 223)	p value	LigaSure™ (n = 132)	Electrosurgery (n = 241)	p value	LigaSure™ (n = 48)	Electrosurgery (n = 67)	p value
**Clinical outcomes**									
Blood loss (mL)	143.6 ± 128.9	154.5 ± 221.7	0.654	180.9 ± 153.7	175.5 ± 161.9	0.753	299.2 ± 240.3	253.8 ± 232.0	.311
Hemoglobin drop (mg/dL)	1.4 ± 0.6	1.2 ± 0.7	0.160	1.3 ± 0.8	1.5 ± 0.8	0.101	2.0 ± 0.8	1.8 ± 0.9	.200
Blood transfusion	1 (2.22%)	11 (4.93%)	0.423	9 (6.82%)	11 (4.56%)	0.355	8 (16.67%)	9 (13.43%)	.630
Complication	0	4 (1.79%)	0.365	1 (0.76%)	5 (2.07%)	0.334	1 (2.08%)	2 (2.99%)	.765
Postop fever		0		1	4		1	0	
Gastrointestinal tract		1		0	1		0	0	
Urinary tract		2		0	0		0	0	
Uterine hematoma		0		0	0		0	1	
Subcutaneous ecchymosis		1		0	1		0	1	
**Efficiency outcomes**									
Operating time (min)	120.6 ± 47.2	97.3 ± 49.1	0.004	109.3 ± 41.3	99.8 ± 45.3	0.047	121.6 ± 41.8	121.7 ± 45.0	.990
Postop stay (d)	2.2 ± 0.5	2.5 ± 0.8	<0.001	2.1 ± 0.4	2.5 ± 0.9	<0.001	2.2 ± 1.0	2.6 ± 0.6	.040
Hospital charges (NTD)	42251.9 ± 13632.1	22793.8 ± 11891.7	<0.001	41066.7 ± 12852.6	24688.7 ± 10464.9	<0.001	44517.7 ± 11077.1	27245.0 ± 10893.7	<.001

Abbreviations: NTD, new Taiwan dollar, Postop = postoperative

Data are reported as mean (standard deviation) or number (%)

There was a slightly higher complication incidence in the LME group, although the difference was not statistically significant. Five (0.66%) women developed low-grade febrile morbidity (< 38.5°C; two in the LML group, three in the LME group). All five patients fully recovered after fluid supplement and intravenous administration of gentamicin 60 mg every 8 h and cefamezine 1 g every 6 h for 1–3 days. Two (0.26%) women in the LME group had postoperative ileus that subsided spontaneously in 2 days after flatus passage, while two (0.26%) in the LME group had acute urinary retention that recovered after 48 h of indwelling catheterization. One (0.13%) woman in the LME group had hematoma formation in the uterus. The patient received oral antibiotic therapy (cephalosporin 500 g every 6 h) for 7 days and regression of the hematoma was confirmed by ultrasonography 3 months postoperatively. Three patients (0.40%) in the LME group developed subcutaneous ecchymosis at the left ancillary cannula site, which subsided spontaneously after 2 weeks. A tissue morcellator was used to remove the specimens in 313 patients: 175 in the LME group and 138 in the LML group. In the rest, the specimens were smoothly removed through the posterior colpotomy.

To reduce the selection-bias effect of comparison between LME and LML groups, propensity score matching was performed for 374 patients, a 1:1 matching method stratified into 187 in LME and 187 patients in LML. Matching variables were all the variables listed in **Table 1. Table 3** shows the comparisons of clinical and efficiency outcomes based on the matched cohort. The mean operating duration did not differ between groups, although the duration was slightly longer in the LME group. The mean postoperative stay was significantly shorter in the LML group than in the LME group. The complication rate was less in the LML group but was not statistically significant. Hospital expenses were significantly higher in the LML group than in the LME group.

**Table 3:** Propensity score 1–1 matched comparison of outcomes after laparoscopic myomectomy with an electrothermal bipolar vessel sealing device (LigaSure™) versus electrosurgery.

	LigaSure™ (n = 187)	Electrosurgery (n = 187)	p value
**Clinical outcomes**			
Fibroids removed (no.)	3.53±3.25	3.23±3.68	.405
Main fibroid size (cm)	7.84±1.98	7.64±2.38	.364
Blood loss (mL)	182.62±162.89	212.99±242.61	.156
Hemoglobin decrease (mg/dL)	1.44±0.78	1.43±0.79	.896
Blood transfusion	8(16.67%)	9(13.43%)	.630
Complication	1(2.08%)	2(2.99%)	.765
**Efficiency outcomes**			
Operating time (min)	109.09±40.89	114.44±51.66	.268
Postop stay (d)	2.10±0.46	2.57±0.91	<.001
Hospital charges (NTD)	41219.44±12862.03	24736.32±10318.09	<.001

Abbreviations: NTD, new Taiwan dollar.

Data are reported as mean (standard deviation) or number (%)

## Discussion

Compared with laparotomy myomectomy, the clear advantages of LM include lesser analgesic use and shorter hospital stay [8]. Other advantages include a lesser decline in hemoglobin concentrations, less abdominal wall trauma, and a faster postoperative recovery [2]. One meta-analysis reported a total increase in time for LM of 13 minutes compared to that for abdominal myomectomy [9]. Another prospective multicenter study of 2050 women showed that the operating time of LM was positively correlated with an increasing dominant myoma diameter [10].

This study showed a favorable result for operating time of conventional diathermy in LM for small and medium myomas. However, this advantage was not seen in patients with large myomas. That is, the LigaSure™ is better suited to LM for large myomas. This might be explained by the properties of the LigaSure™ (i.e., vessel sealer, divider) that facilitate myoma excision. Theoretically, the larger surface area of the myoma pseudocapsule, the longer it will take to complete its excision. Based on our experience, the frequent exchanging of hemostasis and dissection equipment would potentially slow the tumor excision process; this disadvantage would be more prominent in cases of larger tumors. The LigaSure™ simultaneously accomplishes hemostasis and dissection, thus facilitating the excision process. The LigaSure™ is also more capable of managing the vessels supplying greater-diameter myoma. For small and intermediate myomas, these features were diminished due to the smaller surface area of the myoma pseudocapsule and smaller feeding vessel caliber.

During LM for smaller myomas, surgeons tend to omit the hemostasis step if no major bleeding occurs to shorten the time between excision and the subsequent uterine defect repair. Higher blood loss observed in LM by conventional electrosurgery in the small myoma group (Table 2), although not a statistically meaningful finding, may be partially explained by this strategy. In contrast, patients with LML were more likely to undergo GnRH agonist pretreatment in this study. GnRH agonist provided the advantages of shrinking myoma size, but it may enhance surgical difficulty by blurring the myoma margin [11]. This predisposed difficulty may prolong surgical time and affect the operative blood loss in the LML group compared with the LME group for intermediate to large myomas; nevertheless, the difference was not statistically significant.

The application of electrosurgical devices in laparoscopy inevitably results in varying degrees of thermal spread [12–14]. Thermal spread can theoretically damage the adjacent organs (e.g., ureter, bladder, or bowel, or adjacent sensitive structures, particularly nerves) [15]. Evidence has shown that the use of a monopolar electrode compared with other electrosurgical devices resulted in the highest temperatures and the greatest degree of thermal spread in tissues [12, 13]. One of the limitations of our study is that we did not compare the groups in terms of postoperative pain scores or voiding patterns to identify any differences in pain and urinary problems. However, the fact that postoperative hospital stay was universally shorter in the LigaSure™ group might reflect that the potentially lower amount of thermal collateral/proximity damage could be attributed to the LigaSure™ system.

Higher hospital expenses were observed in the LigaSure™ group despite main myoma size. The instrument’s cost would outweigh its benefit of shortening patient hospital stay. This difference was a reasonable consequence of the non-costly hospital stay covered by our public health insurance, while the LigaSure™ had paid for itself by this time point.

## Conclusions

We observed a significantly shorter hospital stay on using the LigaSure™ than on conventional electrosurgery. Conventional electrosurgery is more effective for managing small to medium myomas, whereas the use of the LigaSure™ reduces operation time in cases of myomas ≥10 cm. Thus, LigaSure™ may offer a safe and effective alternative for patients undergoing LM for larger uterine myomas. However, statistical bias should be of concern owing to the characteristics of this retrospective case-control study. Larger prospective studies are warranted to clarify the effectiveness of LigaSure™ in LM.

## Ethics statement

The IRB or ethics committee waived the requirement for informed consent in this retrospective research.

## Supporting information

S1 Table Patient list with all parameters. (XLS)
